# Toxic Indole Diterpenes from Endophyte-Infected Perennial Ryegrass *Lolium perenne* L.: Isolation and Stability

**DOI:** 10.3390/toxins11010016

**Published:** 2019-01-03

**Authors:** Priyanka Reddy, Myrna A. Deseo, Vilnis Ezernieks, Kathryn Guthridge, German Spangenberg, Simone Rochfort

**Affiliations:** 1 Agriculture Victoria, AgriBio, Centre for AgriBioscience, Bundoora, Victoria 3083, Australia; myrnadeseo@gmail.com (M.A.D.); vilnis.ezernieks@ecodev.vic.gov.au (V.E.); kathryn.guthridge@ecodev.vic.gov.au (K.G.); german.spangenberg@ecodev.vic.gov.au (G.S.); 2 School of Applied Systems Biology, La Trobe University, Bundoora, Victoria 3083, Australia

**Keywords:** mycotoxins, terpendole, lolitrem, neurotoxin, alkaloid, endophyte

## Abstract

The most potent of the indole diterpenes, lolitrem B, is found in perennial ryegrass (*Lolium perenne* L.) infected with the endophyte *Epichloë festucae* var. *lolii* (also termed *Lp*TG-1). Ingestion causes a neurological syndrome in grazing livestock called ryegrass staggers disease. To enable the rapid development of new forage varieties, the toxicity of lolitrem B and its biosynthetic intermediates needs to be established. However, most of these indole diterpenes are not commercially available; thus, isolation of these compounds is paramount. A concentrated endophyte-infected perennial ryegrass seed extract was subjected to silica flash chromatography followed by preparative HPLC and purification by crystallization resulting in lolitrem B and the intermediate compounds lolitrem E, paspaline and terpendole B. The four-step isolation and purification method resulted in a 25% yield of lolitrem B. After isolation, lolitrem B readily degraded to its biosynthetic intermediate, lolitriol. We also found that lolitrem B can readily degrade depending on the solvent and storage conditions. The facile method which takes into consideration the associated instability of lolitrem B, led to the purification of indole diterpenes in quantities sufficient for use as analytical standards for identification in pastures, and/or for toxicity testing in pasture development programs.

## 1. Introduction

Perennial ryegrass (*Lolium perenne* L.), is used for forage in temperate regions throughout the world including Northern Europe, the Pacific North West of USA, Japan, South-Eastern Australia and New Zealand [1,2,3,4,5]. It is the most utilized pasture grass on dairy farms in Australia and has a high economic importance [6]. Perennial ryegrass is commonly infected with the fungus *Epichloë festucae* var. *lolii* (also termed *Lp*TG-1) that provides the grass with both abiotic and biotic stress tolerances. Since the 1980s it has been known that this association leads to the production of the indole diterpene lolitrem B that causes ryegrass staggers disease [7]. Lolitrem B was found to be the most abundant and potent of the indole diterpenes produced by *Lp*TG-1 in symbiotic association with perennial ryegrass [8,9]. For forage grass improvement programs, the selection of novel endophytes that produce indole diterpenes that are safe for animals while maintaining the agronomic benefits of having an endophyte, such as insect resistance, is essential.

Targeted screening assays for toxic indole diterpene alkaloids and bioprotective compounds are required to enable better selection of novel endophytes. However, it is a challenge to study these compounds effectively as they are very difficult to isolate and there have been limited studies on the synthesis of the indole diterpenes [10]. Many of the lolitrems and their derivatives have been isolated and the structures elucidated [9,11,12,13,14,15,16,17,18,19]; however, the biological activity remains unknown for many of them. Also, several intermediate compounds along the indole diterpene pathway are not commercially available and the lolitrems in particular show little or no expression in culture but are highly expressed *in planta*. Thus, isolation from perennial ryegrass has been the key source of these compounds [20,21]. The availability of these compounds is essential to undertake further mechanistic studies on their toxicity and mode of action.

The first isolation of lolitrem B was carried out by Gallagher et al., which led to the purification of small quantities of lolitrem B using bioactivity-guided isolation and purification steps over several months [7,17] yielding 15% recovery of lolitrem B. Since the concentration of these alkaloids are generally higher in ryegrass seeds than the ryegrass plant itself [17,22], Miles et al. were able to carry out large scale purification of ryegrass seeds resulting in a 34% yield of lolitrem B using several complex partition and chromatographic steps over a few weeks [17]. Grancher et al. also carried out large scale isolation and purification using a novel counter current chromatography method and multiple steps, resulting in a 32% yield of lolitrem B [23].

In this study, a rapid purification approach was developed to isolate a series of indole diterpenes from a crude hexane extract as required. A crude extract was the preferred starting material of the indole diterpenes, as compounds in natural product extracts are known to increase long-term compound stability [24]. Lolitrem B has been reported to be unstable in acidic conditions [15], but the long-term stability of lolitrem B and its intermediate compounds were not known. Here, we were able to develop an isolation scheme that led to the purification of lolitrem B, lolitrem E [17], paspaline [25] and terpendole B [26] in a short period of time. Compound identity was confirmed by mass spectrometry and NMR spectroscopy in comparison with database or published literature. In this study, it was found that lolitrem B readily degraded to the compound lolitriol [16], which was previously reported to be a biosynthetic intermediate of lolitrem B in the indole diterpene pathway [27]. We also found that lolitrem B can readily degrade depending on the solvent and storage conditions. The isolation scheme developed in this study could easily be repeated to obtain greater quantities of compounds and at the same time could be adapted for the isolation of other indole diterpenes.

## 2. Results

**Figure 1:**
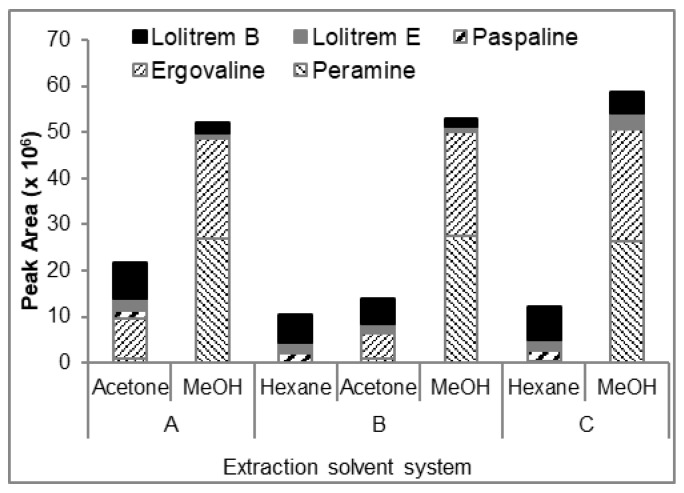
Sum of peak areas from the extracted ion chromatogram of each compound showing the distribution of compounds in the three solvent systems. An ion extraction window of ±0.02 *m*/*z* was used.

A preliminary study was carried out using a combination of solvents for extraction (hexane, acetone and methanol (MeOH)) to determine the optimum solvent system that can be used for the selective extraction of the lipophilic alkaloids, lolitrem B and its biosynthetic intermediate compounds (lolitrem E, lolitriol, paspaline and terpendole B). The extracts were qualitatively analyzed by liquid chromatography-mass spectrometry (LCMS) in triplicate. Since the more polar alkaloids such as peramine and ergovaline are also present in perennial ryegrass-endophyte associations [28], these compounds were included in the preliminary assessment of the LCMS profiles to determine the selectivity of the solvent(s) for the lipophilic alkaloids. The exact mass of the molecular ion of lolitrem B, lolitrem E, paspaline, peramine and ergovaline (Table 1) was used to assess the presence of the metabolites from the total ion chromatograms (TIC) of the extracts using extracted ion technique. The peak area of the target metabolites in the extracted ion chromatogram (EIC) was integrated and the responses were compared (Figure 1). In the sequential extraction with acetone-MeOH (solvent system A), all target metabolites were extracted in both acetone and MeOH at varying proportions. The sequential extraction with hexane-acetone-MeOH (solvent system B) indicated the extractability of most of lolitrem B, lolitrem E and paspaline in hexane and acetone, but acetone was also able to extract ergovaline and peramine. The sequential extraction with hexane-MeOH (solvent system C) showed that the bulk of lolitrem B and paspaline were extracted in hexane [17] and lolitrem E was extracted in both hexane and MeOH. Peramine and ergovaline were selectively extracted in MeOH and thus, solvent system C would be the best choice to carry out the large-scale extraction, whereby the bulk of lolitrem B and its intermediates would be in the hexane extract, and the other alkaloids (if of interest) could be extracted subsequently with MeOH.

**Figure 2:**
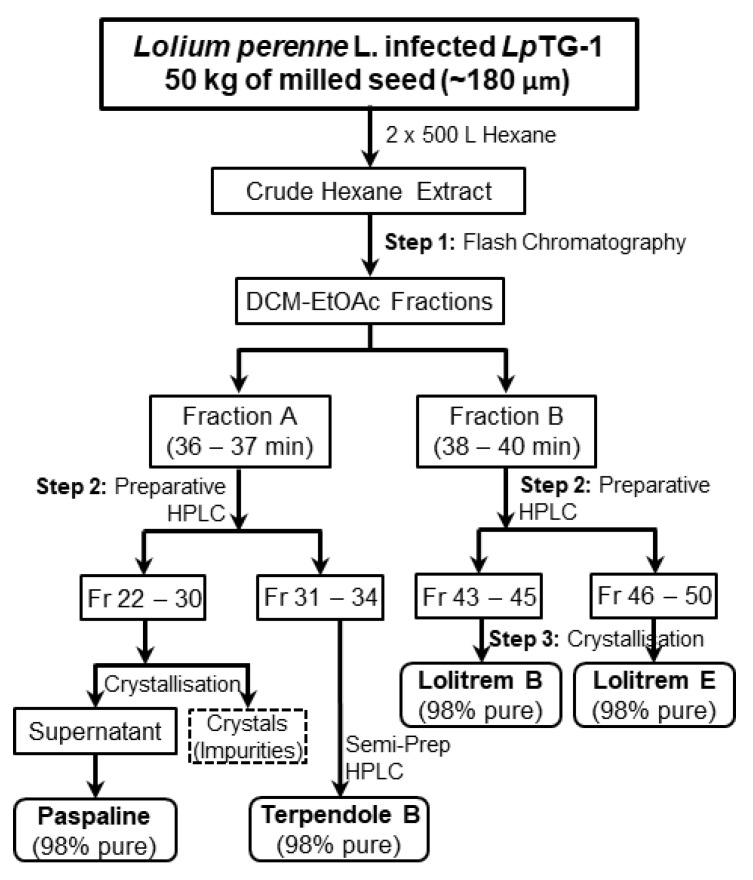
Isolation scheme of paspaline, terpendole B, lolitrem B and lolitrem E from *Lp*TG-1 infected perennial ryegrass seed hexane extract. The solvents DCM and EtOAc refer to dichloromethane and ethyl acetate respectively.

The large-scale extraction was carried out using a sample-to-solvent ratio of 1:10 (*w*/*v*); i.e., 50 kg of seeds with 2 × 500 L of hexane. The preliminary study showed that a second extraction of hexane was necessary as it contributed to 30% of the total lolitrem B. The concentrated crude hexane extract was subjected to fractionation steps (Figure 2) to obtain lolitrem B and its intermediate compounds. The use of the Reveleris flash chromatography system allowed efficient use of solvent and time for the initial chromatographic separation of the crude extract. This step could be repeated several times to obtain enriched fractions for the purification of targeted indole diterpenes.

### 2.1. Lolitrem B and Lolitrem E

Lolitrem B fraction was obtained by preparative High performance liquid chromatography (HPLC) separation of Fraction B from the flash chromatography fractionation (Figure 2) and LCMS and ^1^H NMR spectroscopic analysis (in deuterated chloroform) confirmed the presence of lolitrem B in this fraction (Fr B43–B45), which was allowed to crystallize out of solution to obtain 98% purity by ^1^H NMR spectroscopy and mass spectrometry (Figures S1 and S2). The three-step isolation and purification method resulted in a 25% yield of lolitrem B (1.5 mg/kg of seed) from endophyte-infected perennial ryegrass seed containing 6 mg/kg lolitrem B.

Repeated fractionation by flash chromatography and preparative HPLC to obtain greater amounts of lolitrem B was carried out and ^1^H NMR analysis indicated the stability of lolitrem B up to this isolation step. Lolitrem B (**1**) was purified from the preparative HPLC fraction by crystallization and after analysis by ^1^H NMR spectroscopy, it was found that lolitrem B in its pure form spontaneously degraded while in deuterated chloroform solvent. This was interesting new information because the degree of instability of lolitrem B had not been reported before. Consequently, ^1^H NMR analysis was carried out using deuterated chloroform that was passed through potassium carbonate to neutralize the acid. Lolitrem B was isolated numerous times using the scheme outlined in Figure 2 and ^1^H NMR spectra were acquired for all fractions. The chemical shift data of the isolated lolitrem B (**1)** was found to be in agreement with what had been reported in the literature [15] (Table S1). Consequently, to minimize the risk of degradation of lolitrem B from exposure to acid present in deuterated chloroform, further NMR spectral analysis was carried out using benzene-d_6_ as solvent (Figure S3).

**Figure 3:**
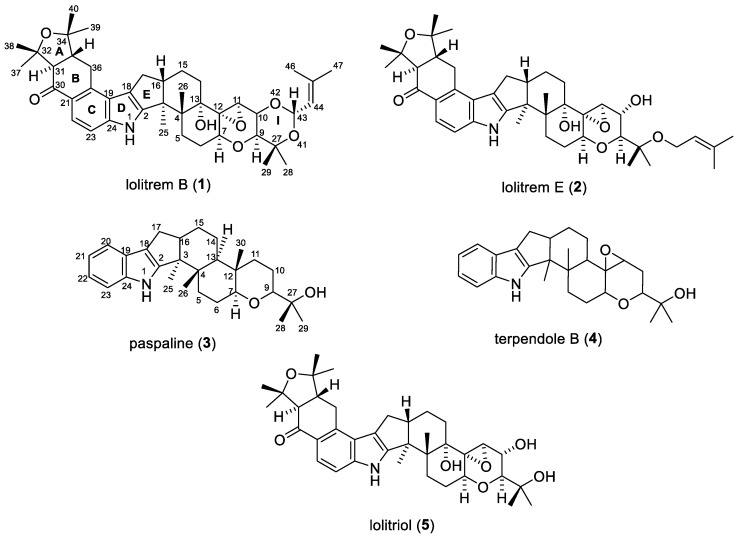
Chemical structures of indole terpene compounds isolated from *Lp*TG-1 infected perennial ryegrass seed and breakdown product of lolitrem B (lolitriol).

Lolitrem E (**2**) was obtained from the fraction eluting after lolitrem B by preparative HPLC and was also purified by crystallization [17]. ^1^H NMR spectroscopy and mass spectrometry (Figures S4 and S5) of the isolated lolitrem E (**2**) indicated 95% purity. The method resulted in 0.2 mg lolitrem E/kg endophyte-infected perennial ryegrass seed. Lolitrem E (**2**) structurally differs from lolitrem B (**1**) from ring I of the latter wherein the ring had opened at the C-42 position (Figure 3). Lolitrem E has been isolated previously from endophyte-infected perennial ryegrass [17].

### 2.2. Paspaline and Terpendole B

The fraction containing paspaline and terpendole B from flash chromatography (Fraction A) was subjected to preparative HPLC that separated paspaline [25] from its analogue, terpendole B (Figure 2). LCMS analysis indicated that the paspaline fraction (Fr A22-A30) contained other components and crystallization resulted in the impurities precipitating out, leaving the supernatant relatively pure with paspaline. ^1^H NMR spectroscopy and mass spectrometry (Figures S6 and S7) of the isolated paspaline (**3**) indicated 98% purity. The ^1^H and ^13^C NMR data are summarized in Table S2 and are in agreement with what had been reported in the literature [25,29].

The preparative HPLC fraction Fr A31–A34 was further subjected to semi-preparative HPLC that yielded terpendole B (**4**), which was confirmed by ^1^H and ^13^C NMR analysis (Table S2). ^1^H NMR spectroscopy and mass spectrometry (Figures S8 and S9) of terpendole B (**4**) indicated 95% purity. Terpendole B was previously isolated by Huang et al [26]. The isolation method resulted in 0.5 mg and 0.2 mg of paspaline (**3**) and terpendole B (**4**) (Figure 3) respectively, per kg of endophyte-infected perennial ryegrass seed.

### 2.3. Lolitrem B Degradation

Lolitrem B rapidly converted to lolitriol (**5**) upon dissolution of lolitrem B in DMSO with sonication. LCMS and NMR spectral analysis (Figures S10 and S11) confirmed the conversion of lolitrem B to lolitriol. Lolitriol has been previously reported with NMR data in 2:1 deuterated chloroform: DMSO [15]. In the present study, NMR spectral data of lolitriol was obtained in deuterated chloroform, as summarized in Table S1.

Lolitrem B in deuterated chloroform was found to spontaneously degrade. NMR spectral analysis confirmed the changes in the chemical structure of what lolitrem B should have been. Since deuterated chloroform can become acidic over time, it is possible that the presence of acid caused the degradation. 

## 3. Discussion

The procedure described here resulted in the concurrent isolation of lolitrem B (**1**), lolitrem E (**2**), paspaline (**3**) and terpendole B (**4**). The endophyte-infected perennial ryegrass seeds extracted contained 6 mg lolitrem B/kg seed and yielded 1.5 mg/kg of lolitrem B (25% yield). The reported quantities of lolitrem B in ryegrass seed can vary significantly [30]. Thus, extraction and isolation methods described in this study can be applied to seeds containing higher levels of lolitrem B (11–13 mg/kg) and its biosynthetic intermediates, to allow greater yields to be isolated. Although methods described by Miles et al. [17] and Grancher et al. [23] have been shown to achieve better yields (32–34% yield), both methods used ryegrass seeds with high lolitrem B content (11–13 mg/kg) and a significant number of complex isolation steps and procedures for the targeted purification of lolitrem B only [17,23].

The simplified method described herein also allowed the stability of the major neurotoxin lolitrem B to be investigated. In deuterated chloroform lolitrem B was found to easily degrade, most probably due to the acidic condition of the solvent. Chloroform in the presence of O_2_ and light turns into phosgene (COCl_2_) and HCl [31]. Typically, alkaloids containing secondary amines have been reported to react with phosgene, incorporating a carbonyl group (C=O) to the molecule [31]. NMR spectral analysis indicated the presence of an additional carbonyl carbon signal in lolitrem B that had degraded in deuterated chloroform and the reaction of lolitrem B with phosgene would probably explain the formation of the breakdown product. Previous reports propose that acid catalyzed hydrolysis of 85% pure lolitrem B results in complete conversion to lolitriol (**5**) [16]. In the present study, it was found that 98% pure lolitrem B could rapidly convert to lolitriol (**5**) upon dissolution of lolitrem B in DMSO with sonication. LCMS and NMR analysis revealed complete conversion of lolitrem B to lolitriol.

Lolitriol (**5**) was reported as a naturally occurring constituent of endophyte-infected ryegrass [16] and LCMS analysis of the hexane extract of ryegrass seed used in this study confirmed its presence in the extract. Given that lolitrem B can easily degrade while in solution, it is also possible that lolitriol could be an artefact and not an intermediate in the biosynthetic pathway [20], depending on the process used for the extraction and isolation of lolitrem B. The observations in this study thus brings in doubt whether lolitriol is naturally occurring or a breakdown product of lolitrem B as a result of the extraction and isolation procedure. However, there is a brief mention of lolitriol being reported to be produced in very low levels from cultures of the fungus *Lp*TG-1 [16]. Further observations during our study confirmed the instability of lolitrem B whereby it degraded while stored in acetonitrile at 4 °C after 4 weeks and this was verified by LCMS analysis. However, lolitrem B was stable when stored in 80% methanol at −20 °C for over 24 months.

Paspaline (**3**) a key intermediate in the biosynthesis of lolitrem B, is likely to be the first stable indole diterpene intermediate and core molecule that is enzymatically altered for the generation of the indole diterpene chemical diversity in various species [21]. Paspaline is not commercially available, although its total synthesis was recently published [10]. It has also been purified and crystallized from other species such as *Penicillium paxilli* and *Claviceps paspali* [25,29]. Terpendole B (**4**) is structurally similar to paspaline (Figure 3) and has been purified from *Albophoma yamanshiensis;* however its biological activity has never been reported in mammals.

The amounts and purity of the compounds isolated are sufficient for in vitro and small mouse in vivo studies, as well as LCMS analytical standards. Also, the proposed isolation method is time efficient, as only a maximum of four steps are required for the purification of one compound along the indole diterpene pathway. The method will also allow purification of other intermediates within the indole diterpene pathway that are not commercially available.

## 4. Materials and Methods

### 4.1. Materials

Perennial ryegrass seed of cultivar Victorian Perennial was obtained from Heritage Seeds, Dandenong South, Victoria, Australia. The 50 kg of seed used for the large-scale extraction was ground in a clean mill facility at Propharma Australia, Dandenong South, Victoria, Australia using a multi swing hammer mill and a 180 µM sieve.

### 4.2. Preliminary Extraction

Sequential extraction using three different solvent systems were tested: (A) acetone-methanol; (B) hexane-acetone-methanol; and (C) hexane-methanol. Fifty grams of ground seeds were each weighed into three separate 1 L flasks with stir bars and 500 mL of the first solvent was added. The headspace of the flasks was flushed with nitrogen gas and then stirred on a magnetic stirrer—hot plate at room temperature for 5 h. The first extracts were each carefully transferred into separate clean flasks and a second volume (500 mL) of the same solvent was added into the flasks with the seed residue and a second extraction with same solvent was repeated for 16 h with stirring at room temperature. The second extracts were combined with their respective first extracts and concentrated to dryness in a rotary evaporator. The sample procedure (extraction volumes and times as well as pooling procedure) was applied/repeated with the second extraction solvent. A similar process was used for solvent system B with methanol as the third solvent. The dried hexane and acetone extracts were reconstituted in 1 mL of methanol-dichloromethane (1:1) and the dried methanol extracts were resuspended in 1 mL of methanol. A 10 µL aliquot of each sample was diluted 100-fold with methanol and transferred into a vial for LCMS analysis.

### 4.3. Large Scale Extraction

Fifty kg of ground seed (containing 6 mg lolitrem B/kg seed) was extracted with 500 L of hexanes (1:10 sample-solvent ratio) with stirring for 5 hours in a 630 L jacketed reactor housed at a commercial custom chemical synthesis facility (Boron Molecular, Noble Park, Victoria, Australia). The first hexane extract was collected (Hexane 1) and a second extraction of the sample residue was carried out with another 500 L of hexanes for 18 hours with stirring. The second hexane extract was collected (Hexane 2), combined with Hexane 1 and concentrated to approximately 30 L by solvent distillation and further reduced to approximately 700 mL using a large-scale rotary evaporator.

### 4.4. Silica Flash Chromatography

A slurry of crude hexane extract (10–15 mL), which is equivalent to 1 kg of seed, was mixed with 30 mL of hexane-dichloromethane (2:1) and injected directly into a dry 330 g Reveleris HP 20 µm Silica Cartridge (Grace Discovery Sciences, Epping, Victoria, Australia) which was then fitted to a Reveleris® Flash Chromatography System X2 (Grace Discovery Sciences, Epping, Victoria, Australia) with UV/VIS Detector and evaporative light scattering detector (ELSD). The UV/Vis detector was set to 254 nm, 268 nm and 320 nm wavelengths. Normal phase chromatographic separation was performed by gradient elution using four solvents with a total run time of 60 min. Solvents used were petroleum spirits, 40–60 °C (Ajax Finechem, Analytical Reagent grade) as Solvent A; dichloromethane (DCM) (Burdick & Jackson, HPLC grade) as Solvent B; ethyl acetate (EtOAc) (Ajax Finechem, Analytical Reagent grade) as Solvent C; and methanol (MeOH) (LiChrosolv, HPLC grade) as Solvent D. The three gradient elution steps were as follows:

Gradient 1 (A:B): At a flow rate of 50 mL/min slowly increased over 13 min to 115 mL/min at 3% B, followed by a linear gradient from 3% to 100% B for 12 min and 100% B for 5 min. Gradient 2 (B:C): 0% C to 100% C for 13 min then 100% C for 2 min. Gradient 3 (C:D): 0% D to 100% D for 13 min then 100% D for 2 min. The fractions were analyzed by LCMS and the target compounds were found to be in the 30–40 min region of the chromatogram (30–70% DCM-EtOAc). Fraction A (36–37 min) contained paspaline and terpendole B and fraction B (38–40 min) contained lolitrem B and lolitrem E.

### 4.5. Preparative HPLC System

A Dionex Ultimate 3000 solvent delivery system (Dionex, Sunnyvale, CA, USA) was used equipped with a binary pump, photodiode array detector (PDA 3000), attached to a Rheodyne Model 7725 injector with a 1 mL injector loop and operated using Chromeleon version 6.8 software (Dionex, Sunnyvale, CA, USA). The mobile phases used were DCM (Burdick & Jackson, HPLC grade) as mobile phase A and acetonitrile (LiChrosolv, HPLC grade) as mobile phase B at a flow rate of 12 mL/min, using a Phenomenex Luna 10 µm Silica (2) 100Å, 250 × 21 mm column [11,17,18].

### 4.6. Lolitrem B (***1***) and Lolitrem E (***2***)

Fraction B, that contained lolitrem B and lolitrem E, was evaporated to dryness and resuspended in 1 mL of 50:50 DCM/ACN and subsequently subjected to preparative HPLC as shown in Figure 2. Initial conditions were 5% B (95% A) for 10 min then a linear gradient to 8% B over a period of 30 min and to 10% B over a period of 20 min. The fractions generated were analyzed using LCMS and it was found that lolitrem B eluted at a retention time of 36 min and lolitrem E eluted at 40 min [17]. The preparative HPLC fractions that contained lolitrem B (Fr B43–B45) and lolitrem E (Fr B46–B50) were each concentrated in separate 4 mL vials using a stream of N_2_ gas and were dissolved in minimal quantities of dichloromethane followed by an equal volume of acetonitrile. A perforated cap was placed on the vials, placed in a 4 °C fridge to allow crystallization. White crystals of lolitrem B (**1**) and lolitrem E (**2**) each formed after 2 days and the crystals were collected and washed with cold acetonitrile.

### 4.7. Paspaline (***3***)

Fraction A from the flash chromatography was subjected to preparative HPLC (Figure 2). Initial conditions were 8% B (92% A) for 5 min then a linear gradient to 10% B over a period of 10 min and held for 30 min, followed by a 20% B over at 30.1 min for a period of 5 min. The fractions were analyzed by LCMS and it was found that paspaline eluted at a retention time of 34 min and terpendole B eluted at 36 min. The fractions that contained paspaline (Fr A22–A30) were concentrated to dryness using a stream of N_2_ gas and transferred to a 4 mL vial using a minimal volume of 1 mL dichloromethane followed by an equal volume of acetonitrile. The solution was allowed to crystallize and paspaline (**3**) remained in the supernatant.

### 4.8. Terpendole B (***4***)

The preparative HPLC fraction containing terpendole B (Fr A31–A34) was further purified by semi-preparative HPLC using an Agilent 1100 Series HPLC (Agilent Technologies, Santa Clara, CA, USA) equipped with quaternary pump, autoinjector and a diode array detector (DAD) using ChemStation B.04.02 software (Agilent Technologies, Santa Clara, CA, USA). Target compound was eluted from a Phenomenex Luna 5 µm Silica (2), 250 × 10 mm column with DCM (Burdick & Jackson, HPLC grade) as mobile phase A and acetonitrile (LiChrosolv, HPLC grade) as mobile phase B at 3 mL/min flow rate. General conditions were 5% B (95% A) for 20 min to 8% B for a period of 30 min and then to 10% B for 30 min. Terpendole B (**4**) eluted at 38 min.

### 4.9. Lolitriol (***5***)

Lolitriol was serendipitously found when lolitrem B that was isolated as above was dissolved in DMSO (Sigma Aldrich, catalogue no. M81802) with repeated sonication. LCMS analysis of the solution showed that the supposed lolitrem B peak eluted earlier than expected and NMR spectroscopic analysis revealed that lolitrem B had degraded to lolitriol.

### 4.10. Liquid Chromatography-Mass Spectrometry (LCMS) Analysis

Pure paspaline, lolitrem E and column fractions were analyzed on an Agilent 1200 series Infinity UHPLC system (Agilent Technologies, Santa Clara, CA, USA) with binary pump, ALS autosampler equipped with an 8 μL loop capillary and temperature-controlled column compartment coupled to an Agilent 6538 UHD Accurate-Mass Q-TOF LC/MS detector equipped with a dual ESI source was used for analysis of extracts and fractions. A Dionex Acclaim RSLC 120 C18 HPLC column. 2.1 × 150, 2.2 µM particle size was used for analysis. Mass spectrometry data was acquired using MassHunter Workstation Software B.05.00 (Agilent Technologies, Santa Clara, CA, USA) and qualitative data analysis was conducted using the MassHunter Workstation Software Qualitative Analysis B.06.00 (Agilent Technologies, Santa Clara, CA, USA).

The MS detector was operated in positive electrospray ionization (ESI+) mode using full-scan with a mass range of *m*/*z* 110–2000. An internal isocratic pump delivered internal reference mass solution with *m*/*z* 121.0508 and 922.0098 (ESI+). The ESI drying gas (N_2_) was set at a flow rate of 7 L/min at 350 °C and nebulizer gas (N_2_) pressure was set at 0.31 MPa. Capillary, fragmentor and skimmer voltage was set at 3500 V, 175 V and 65 V, respectively. Octopole voltage was left at its default value of 750 V. All MS/MS (MS^2^) spectra were acquired on selected samples with a collision energy of 20 and 40 V. Mass spectrometry data was acquired using MassHunter Workstation Software B.05.00 (Agilent Technologies, Santa Clara, CA, USA) and qualitative data analysis was conducted using the MassHunter Workstation Software Qualitative Analysis B.06.00.

Pure lolitrem B, lolitriol and terpendole B fractions were analyzed on a Vanquish liquid chromatography system (Thermo Fisher Scientific, Waltham, MA, USA) with a binary pump, autosampler and temperature-controlled column compartment coupled with a QExactive (QE) Plus mass spectrometer (Thermo Fisher Scientific, Waltham, MA, USA; Thermo, Bremen, Germany) detector. A Thermo Fisher Scientific Hypersil Gold 1.9 µm, 100 mm × 2.1 mm reverse phase column was used. 

The QE mass detector was set at FT positive mode over a mass range of 80–1200 amu with resolution set at 35,000. Nitrogen was used as the sheath, auxiliary and sweep gas at a flow rate of 28, 15 and 4 L/min, respectively and the spray voltage was set at 3600 V (positive) and 3300 (negative). The capillary temperature was set to 300 °C, S-lens RF level set at 64 and an auxiliary gas heater temperature of 310 °C. Mass spectrometry data was acquired using Thermo Xcalibur V. 2.1 (Thermo Fisher Scientific, Waltham, MA, USA) and data was analyzed using Thermo Xcalibur Qual Browser V. 2.1 (Thermo Fisher Scientific, Waltham, MA, USA).

All chromatographic separation was performed by gradient elution using water with 0.1% formic acid (Sigma-Aldrich CHROMASOLV®, Castle Hill, NSW, Australia, HPLC grade) as Solvent A and acetonitrile with 0.1% formic acid (Sigma-Aldrich CHROMASOLV®, Castle Hill, NSW, Australia, HPLC grade ≥99.9%) as Solvent B at a flow rate of 0.3 mL/min. Initial conditions were 98% A before initiating a linear gradient to 100% B over 11 min, and this was maintained for 4 min before returning to the initial gradient conditions. Injection volume was 2 μL.

### 4.11. Nuclear Magnetic Resonance (NMR) Spectroscopy

Samples were dissolved either in deuterated chloroform (CDCl_3_, Cambridge Isotope Laboratories, Inc., Tewksbury, MA, USA) or deuterated benzene (C_6_D_6_, Cambridge Isotope Laboratories, Inc., Tewksbury, MA, USA). 1D and 2D NMR spectra were obtained on a Bruker Avance III 700 MHz spectrometer equipped with a cryoprobe (Bruker CPQCI H-P/C/N-D) and Sample Jet automatic sample changer with cooling (Bruker BioSpin GmbH, Rheinstetten, Germany) using Bruker pulse programs. Data acquisition was performed at 25 °C and processing software was Topspin v.3.2 (Bruker BioSpin GmbH, Rheinstetten, Germany).

## Figures and Tables

**Table 1:** The exact mass [M+H]^+^ and retention time (RT) of alkaloids used to assess the presence of target compounds in extracts and the quantity of alkaloids isolated and purified per kg of seed.

Compound	Molecular Formula	Exact Mass, [M+H]^+^	RT, min	Quantity Purified (mg/kg of Seed)
lolitrem B	C_42_H_55_NO_7_	686.4055	11.1	1.5
lolitrem E	C_42_H_57_NO_7_	688.4213	11.3	0.2
paspaline	C_28_H_39_NO_2_	422.3059	12.0	0.5
terpendole B	C_27_H_35_NO_3_	422.2690	12.2	0.2
ergovaline ^1^	C_29_H_35_N_5_O_5_	534.2709	5.5	-
peramine ^1^	C_12_H_17_N_5_O	248.1506	3.5	-

^1^ These alkaloids were not isolated and purified in this study.

## Supplementary Materials

The following are available online at http://www.mdpi.com/2072-6651/11/1/16/s1, Table S1: ^1^H and ^13^C NMR chemical shifts of lolitrem B (1), lolitrem E (2) and lolitriol (5), (700 MHz, CDCl_3_). Table S2: ^1^H and ^13^C NMR chemical shifts of paspaline (3) and terpendole B (4) (700 MHz, CDCl_3_). Figure S1: ^1^H NMR spectrum of a pure fraction of lolitrem B (700 MHz, CDCl_3_). Figure S2: Mass spectrum of lolitrem B in positive ion mode (ESI+) LC-ESI-FTMS with an observed ion at *m*/*z* 686.4013 [M+H]^+^ (5.5 ppm). Figure S3: ^1^H NMR spectrum of a pure fraction of lolitrem B (700 MHz, Benzene-d_6_). Figure S4: ^1^H NMR spectrum of a pure fraction of lolitrem E (700 MHz, CDCl_3_). Figure S5: Mass spectrum of lolitrem E in positive ion mode (ESI+) LC-ESI-Q-TOF with an observed ion at *m*/*z* 688.4201 [M+H]^+^, (1.74 ppm). Figure S6: ^1^H NMR spectrum of a pure fraction of paspaline (700 MHz, CDCl_3_). Figure S7: Mass spectrum of the paspaline in positive ion mode (ESI+) LC-ESI-Q-TOF with an observed ion at *m*/*z* 422.3059 [M+H]^+^, (0.0 ppm). Figure S8: ^1^H NMR spectrum of a pure fraction of terpendole B (700 MHz, CDCl_3_). Figure S9: Mass spectrum of the terpendole B in positive ion mode (ESI+) LC-ESI-FTMS with an observed ion at *m*/*z* 422.2681 [M+H]^+^ (1.9 ppm). Figure S10: ^1^H NMR spectrum of lolitriol (700 MHz, CDCl_3_). Figure S11: Mass spectrum of lolitriol in positive ion mode (ESI+) LC-ESI-FTMS with an observed ion at *m*/*z* 620.3573 [M+H]^+^ (1.4 ppm).
