# Supplementary material for: Toxic Indole Diterpenes from Endophyte-Infected Perennial Ryegrass Lolium perenne L.: Isolation and Stability
Source: Toxins (Basel). 2019 Jan 3;11(1):16. doi: 10.3390/toxins11010016 (PMC6356652; doi:10.3390/toxins11010016)
Supplement: Supplementary file 1 [file toxins-11-00016-s001.pdf]

Supplementary Materials: Toxic Indole Diterpenes from Endophyte-Infected Perennial Ryegrass *Lolium perenne* L.: Isolation and Stability

Priyanka Reddy, Myrna A. Deseo, Vilnis Ezernieks, Kathryn Guthridge, German Spangenberg and Simone Rochfort

Table S1. ^{1}H and ^{13}C NMR chemical shifts of lolitrem B (**1**), lolitrem E (**2**) and lolitriol (**5**), (700 MHz, $CDCl_3$).

Position	Lolitrem B (1)		Lolitrem E (2)		Lolitriol (5)	
	^{1}H (*J* in Hz)	^{13}C	^{1}H (*J* in Hz)	^{13}C	^{1}H (*J* in Hz)	^{13}C
2		152.8		152.7		152.8
3		50.7		50.7		50.7
4		42.5		42.4		42.4
5a	2.70 *td* (5.3, 14.0)	27.6	2.72 *td* (5.6, 14.0)	27.4	1.32 *m*	27.6
5b	1.35 *m*		1.29 *m*		2.71 *m*	
6a	2.28 *m*	28.1	2.27 *m*	27.7	2.29 *m*	27.9
6b	1.77 *tdd* (5.3, 9.0, 14.0)		1.75 *tdd* (5.6, 9.1, 14.0)		1.75 *tdd* (5.6, 9.0, 14.0)	
7	4.32 *t* (9.0)	71.6	4.18 *t* (9.0)	71.3	4.19 *t* (9.0)	71.5
9	3.56 *d* (9.0)	71.4	3.41 *d* (9.0)	74.7	3.32 *d* (9.0)	76.9
10	3.90 *d* (9.0)	71.2	3.97 *dd* (9.0)	67.1	3.99 *d* (9.0)	67.5
11	3.61 *s*	61.3	3.59 *s*	64.3	3.60 *s*	64.7
12		67.8		68.5		70.0
13		78.2		77.8		78.0
14a	1.56 *dt* (3.0 13.5)	30.4	1.59 *dt* (3.0, 13.5)	30.1	1.58 *d* (13.5)	30.5
14b	1.44 *dd* (3.0, 13.5)		1.48 *m*		1.47 *dt* (3.0, 13.5)	
15a	1.93 *ddd* (3.0, 12.5, 25)	20.6	1.93 *ddd* (3.0, 12.5, 25)	20.4	1.93 *m*	20.5
15b	1.64 *m*		1.63 *m*		1.64 *d* (13.5)	
16	2.84 *m*	50.2	2.85 *m*	50.0	2.85 *m*	50.3
17a	2.62 *dd* (11.0, 13.0)	29.3	2.62 *dd* (11.0, 13.0)	29.1	2.62 *m*	29.3
17b	2.92 *dd* (6.0, 13.0)		2.92 *dd* (6.0, 13.0)		2.92 *d* (13.0)	
18		118.8		118.6		118.8
19		126.1		123.9		126.3
20		124.0		126.0		124.2
21		137.1		137.2		137.2
22	7.86 *d* (8.6)	120.5	7.85 *d* (8.6)	120.2	7.85 *d* (8.6)	120.5
23	7.21 *d* (8.6)	110.5	7.20 *d* (8.6)	110.3	7.20 *d* (8.6)	110.5
24		142.0		141.8		142.2
25	1.27 *s*	16.1	1.27 *s*	15.9	1.27 *s*	16.2
26	1.14 *s*	19.1	1.12 *s*	18.7	1.11 *s*	19.0
27		74.9		78.5		73.8
28	1.289 *s*	28.4	1.27 *s*	24.2	1.23 *s*	24.2
29	1.280 *s*	16.7	1.21 *s*	28.5	1.28 *s*	28.5
30		196.6		196.9		196.7

31	2.76 *d* (14.3)	60.1	2.76 *d* (14.0)	59.9	2.76	60.1
32		80.0		79.8		80.2
34		79.3		79.0		79.5
35	2.65 *m*	50.0	2.66 *m*	49.7	2.65 *m*	50.0
36a	2.95 *dd* (12, 15.3)	28.4	2.96 *dd* (12, 15.3)	28.3	2.95 *dd* (12, 15.3)	28.6
36b	3.42 *dd* (4.1, 15.3)		3.42 *dd* (4.1, 15.3)		3.42 *dd* (4.1, 15.3)	
37	1.52 *s*	30.8	1.52 *s*	30.6	1.52 *s*	30.9
38	1.31 *s*	25.2	1.30 *s*	25.0	1.31 *s*	25.3
39	1.24 *s*	25.2	1.24 *s*	25.3	1.23 *s*	25.3
40	1.38 *s*	29.5	1.37 *s*	29.6	1.37 *s*	29.6
43	5.52 *d* (6.6)	92.8	3.94 *d* (6.7)	57.7		
44	5.29 *d* (6.6)	122.1	5.24 *t* (6.7)	120.6		
45		139.7		137.2		
46	1.71 *s*	18.8	1.69 *s*	17.7		
47	1.73 *s*	25.8	1.64 *s*	25.6		
NH	7.97 br *s*		7.95 br *s*		8.02 br *s*	

br = broad, t = triplet, d = doublet, td = triplet of doublets, tdd = triplet of doublet of doublets, dd = doublet of doublets, dt = doublet of triplets, m = multiplet, s = singlet, ddd = doublet of doublet of doublets

Table S2. ^{1}H and ^{13}C NMR chemical shifts of paspaline (**3**) and terpendole B (**4**) (700 MHz, $CDCl_3$).

Position	Paspaline (3)		Terpendole B (4)	
	**^{1}H (*J* in Hz)**	**^{13}C**	**^{1}H (*J* in Hz)**	**^{13}C**
2		150.7		150.2
3		52.8		50.6
4		39.8		39.9
5a	1.95 *td* (4.0, 13.0)	34.0	1.94 *td* (4.3, 13.6)	32.8
5b	1.60 *m*		1.58 *m*	
6a	1.60 *m*	25.1	1.83 *tdd* (4.3, 10.2, 13.6)	29.6
6b	1.77 *m*		2.25 *m*	
7	3.00 *dd* (3.7, 12.0)	85.5	3.94 *t* (8.1)	73.8
9	3.20 *dd* (2.7, 12.1)	84.5	3.57 *dd* (2.2, 11.1)	74.9
10a	1.62 *m*	21.8	1.78 *dd* (11.1, 14.0)	26.5
10b	1.43 *m*		2.04 br *d* (14.0)	
11a	1.12 *m*	37.5	3.42 *d* (2.0)	56.0
11b	1.82 *m*			
12	-	36.5	-	61.8
13	1.46 *m*	46.3	2.10 *dd* (3.2, 13.0)	41.1
14a	1.37 *m*	21.8	1.66 *m*	22.3
14b	1.67 *m*		1.05 *m*	
15a	1.59 *m*	25.1	1.57 *m*	24.4
15b	1.76 *m*		1.71 *m*	
16	2.75 *m*	48.6	2.76 *m*	49.9
17a	2.31 *m*	27.3	2.68 *dd* (6.3, 13.3)	27.5
17b	2.65 *dd* (6.5, 13.3)		2.34 *dd* (10.5, 13.3)	
18		118.2		118.6
19		125.0		125.4
20	7.40 *m*	118.1	7.41 *d* (7.5)	118.7
21	7.05 *m*	119.3	7.06 *dq* (1.4, 6, 1.9)	120.0

22	7.06 *m*	120.2	7.07 *dq* (1.4, 6, 1.9)	121.0
23	7.27 *m*	111.2	7.29 *d* (7.5)	111.7
24		139.9		140.2
25	1.01 *s*	14.4	1.01 *s*	14.8
26	1.12 *s*	19.8	1.05 *s*	16.7
27		71.8		71.5
28	1.16 *s*	23.7	1.10 *s*	24.1
29	1.17 *s*	26.0	1.16 *s*	26.7
30	0.86 *s*	12.6		
NH (s)	7.67 br *s*		7.72 br *s*	

br = broad, t = triplet, td = triplet of doublets, dd = doublet of doublets, dq = doublet of quartets, m = multiplet, s = singlet.

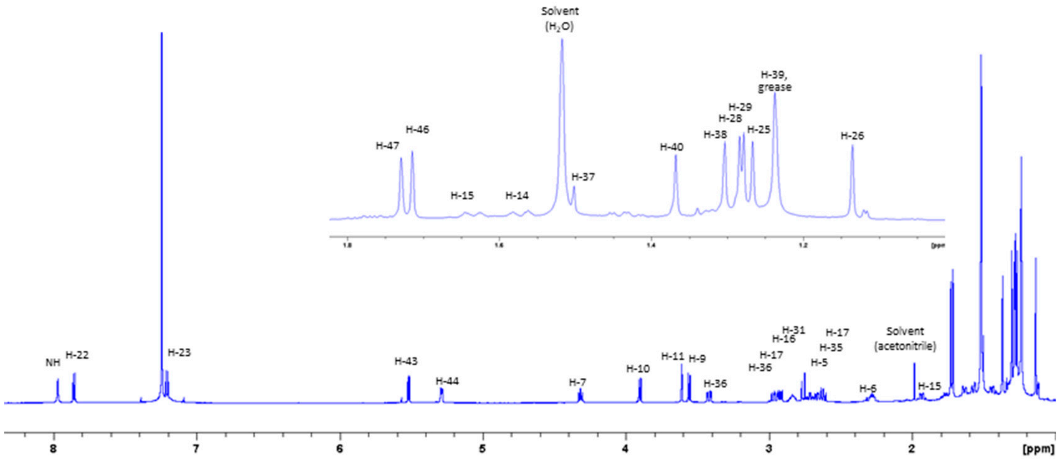

Figure S1. ^{1}H NMR spectrum of a pure fraction of lolitrem B (700 MHz, $CDCl_3$).

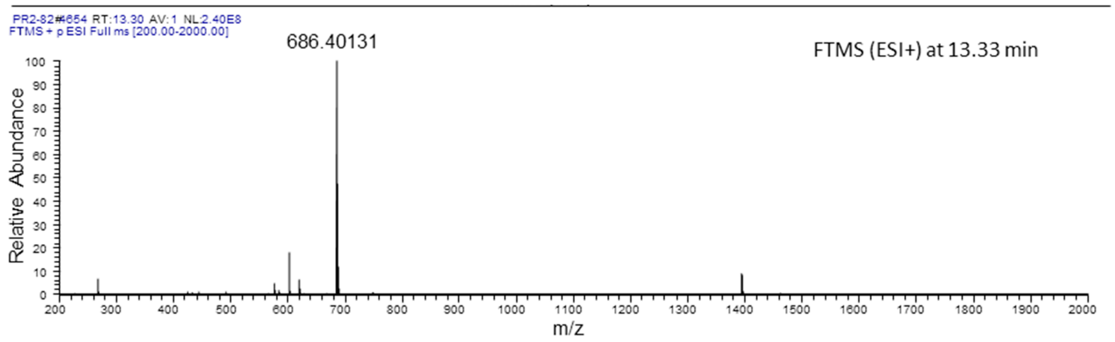

Figure S2. Mass spectrum of lolitrem B in positive ion mode (ESI+) LC-ESI-FTMS with an observed ion at *m*/*z* 686.4013 $[M+H]^+$ (Δ 5.5 ppm).

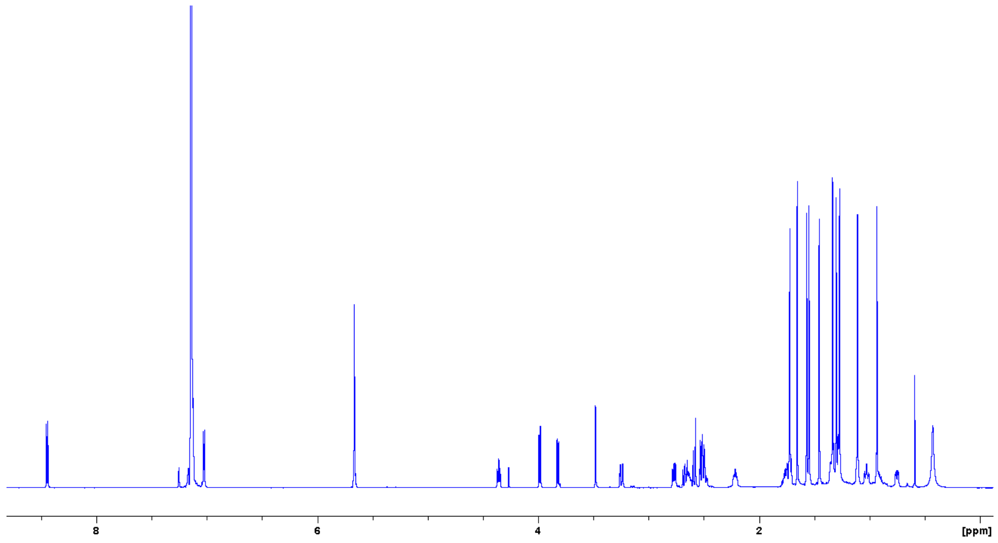

Figure S3. ^{1}H NMR spectrum of a pure fraction of lolitrem B (700 MHz, Benzene-d6).

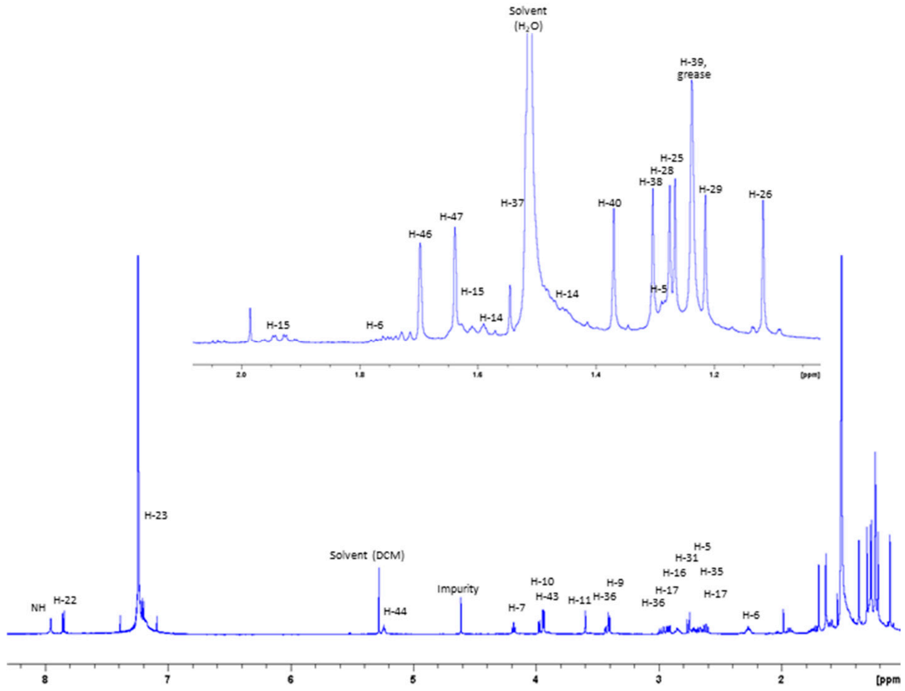

Figure S4. ^{1}H NMR spectrum of a pure fraction of lolitrem E (700 MHz, $CDCl_3$).

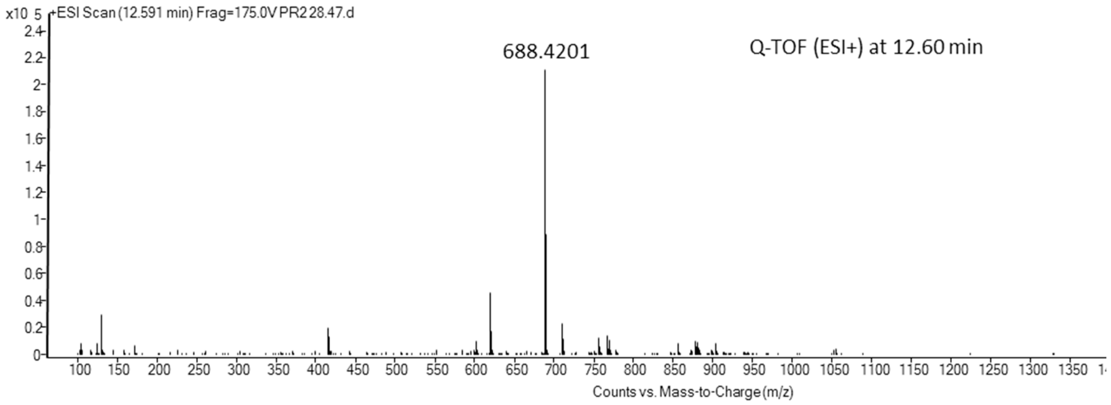

Figure S5. Mass spectrum of lolitrem E in positive ion mode (ESI+) LC-ESI-Q-TOF with an observed ion at *m*/*z* 688.4201 [M+H]$^+$ (Δ 1.74 ppm).

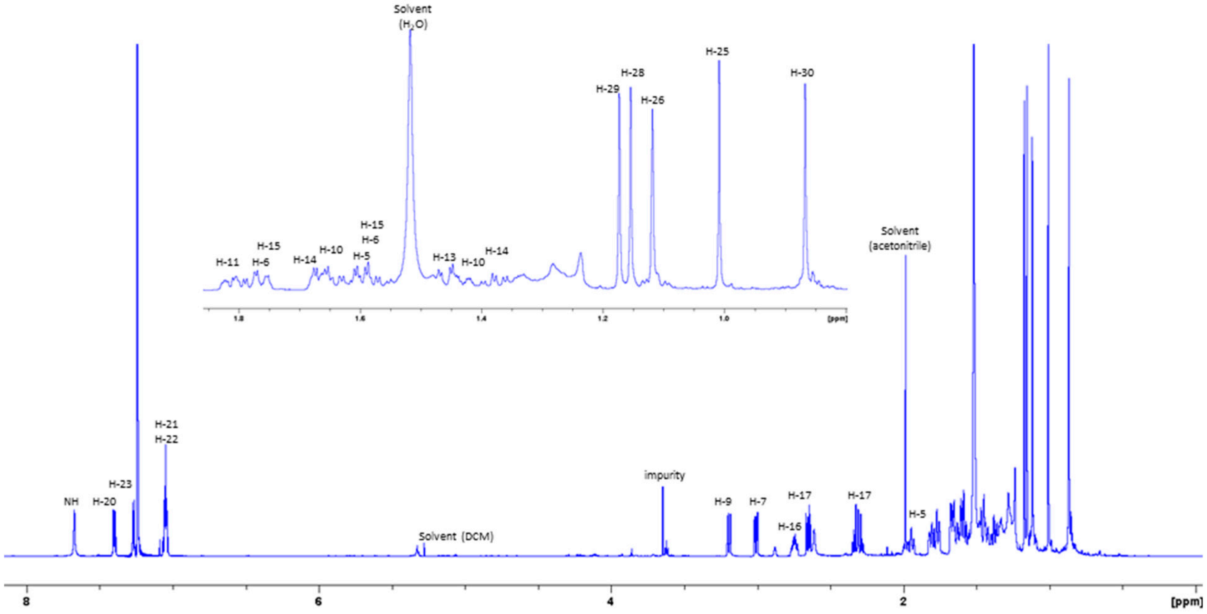

Figure S6. ^{1}H NMR spectrum of a pure fraction of paspaline (700 MHz, $CDCl_3$).

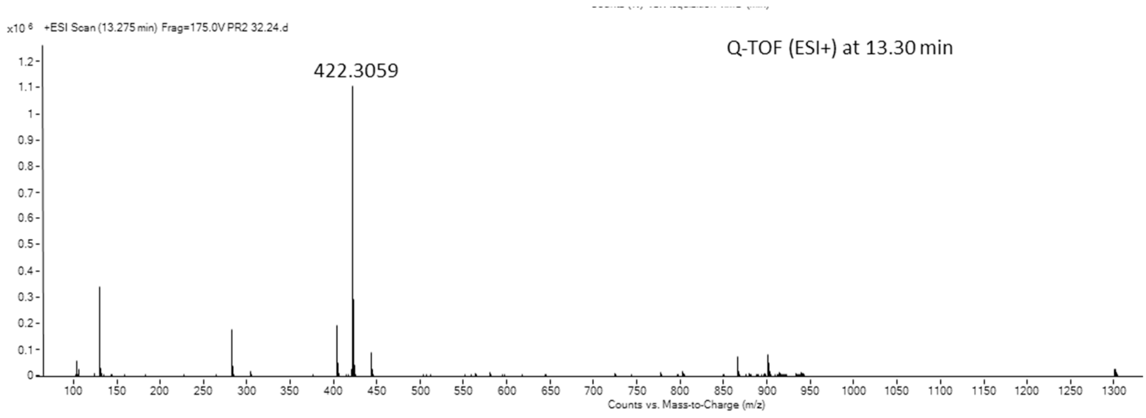

Figure S7. Mass spectrum of the paspaline in positive ion mode (ESI+) LC-ESI-Q-TOF with an observed ion at *m*/*z* 422.3059 [M+H]$^+$ (Δ 0.0 ppm).

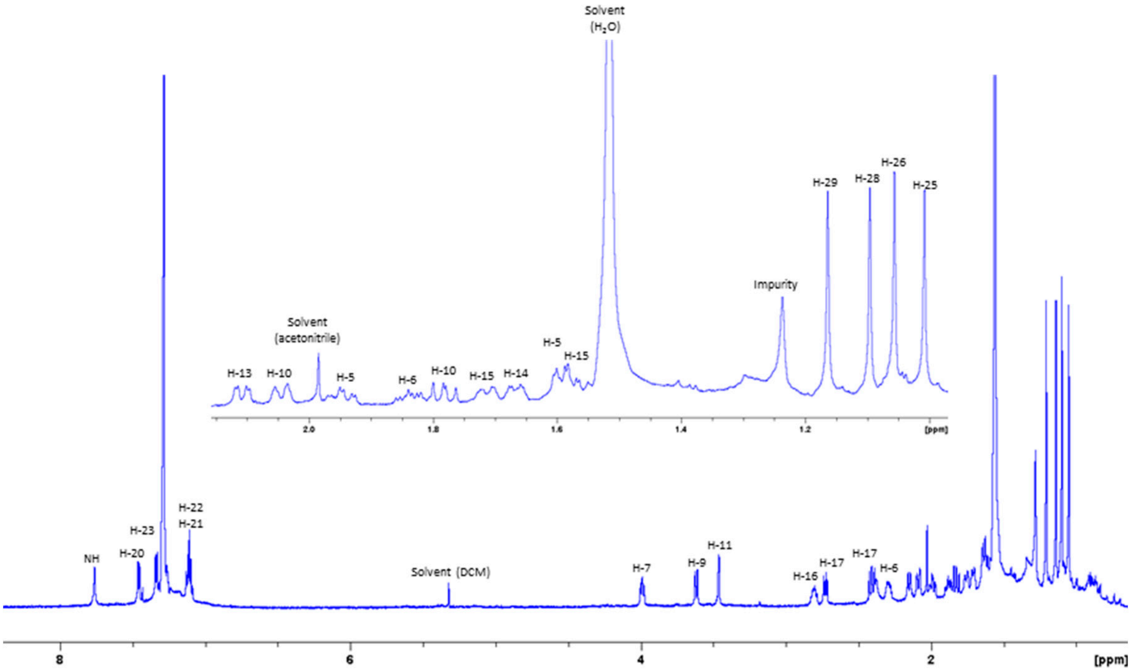

Figure S8. ^{1}H NMR spectrum of a pure fraction of terpendole B (700 MHz, $CDCl_3$).

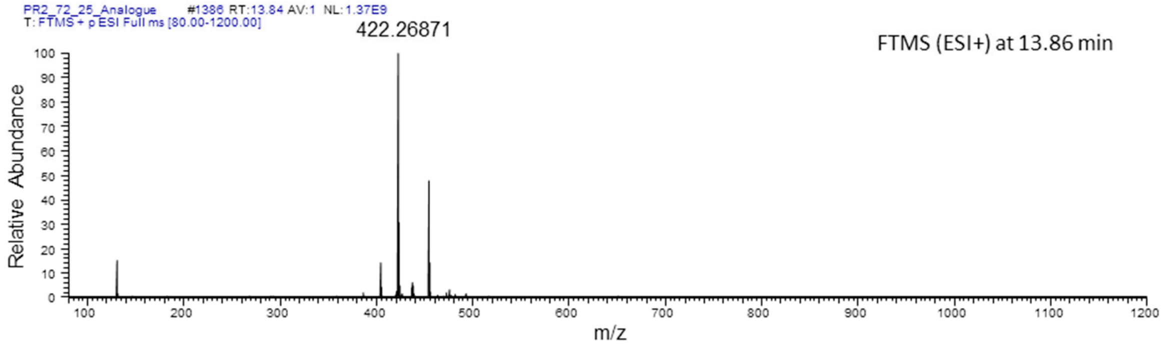

Figure S9. Mass spectrum of the terpendole B in positive ion mode (ESI+) LC-ESI-FTMS with an observed ion at m/z 422.2681 [M+H]+ (Δ 1.9 ppm).

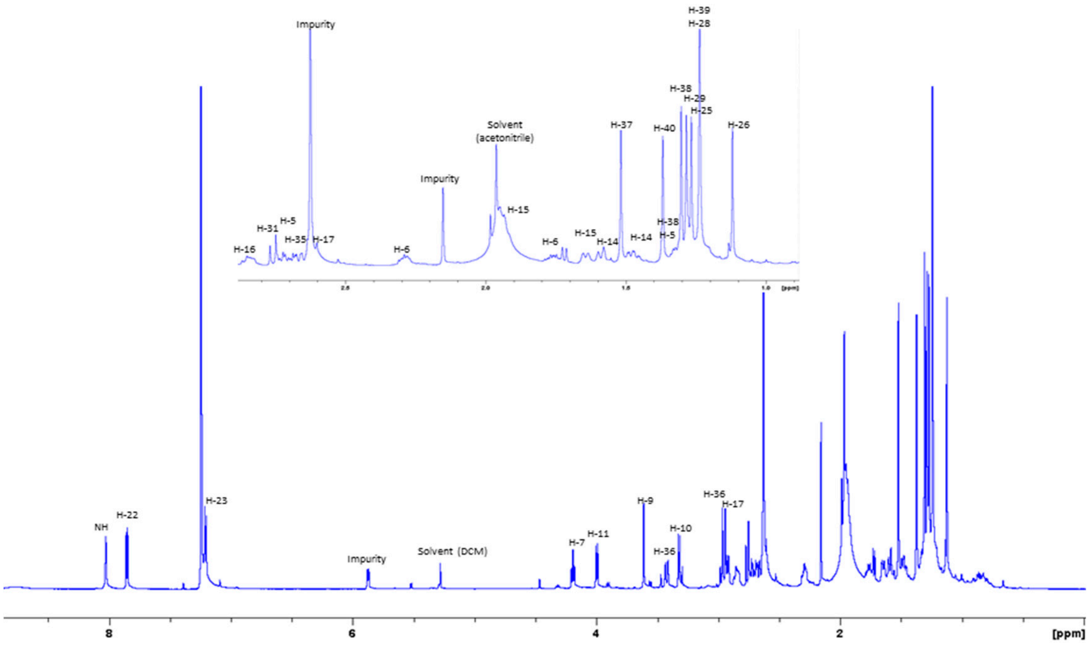

Figure S10. ¹H NMR spectrum of lolitriol (700 MHz, $CDCl3$).

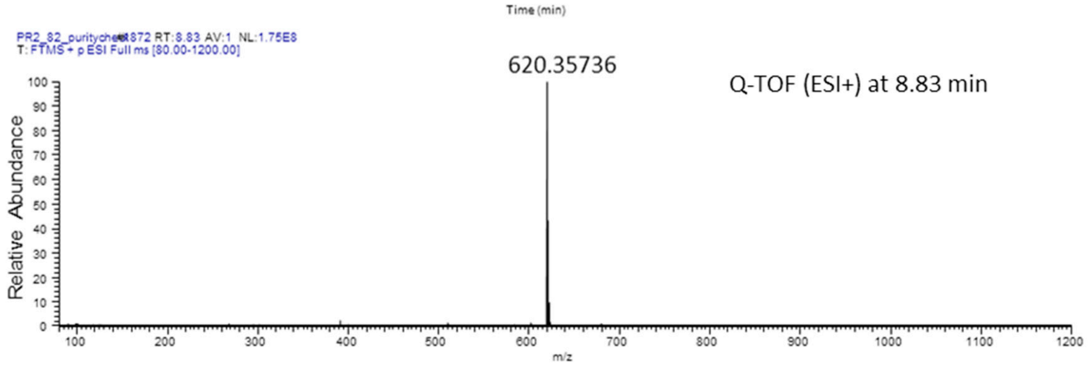

Figure S11. Mass spectrum of lolitriol in positive ion mode (ESI+) LC-ESI-FTMS with an observed ion at *m*/*z* 620.3573 $[M+H]^+$ (Δ 1.4 ppm).
